# Quantum Graph Neural Network Models for Materials Search

**DOI:** 10.3390/ma16124300

**Published:** 2023-06-10

**Authors:** Ju-Young Ryu, Eyuel Elala, June-Koo Kevin Rhee

**Affiliations:** 1 School of Electrical Engineering & ITRC of Quantum Computing for AI, KAIST, 291 Daehak-ro, Yuseong-gu, Daejeon 34141, Republic of Korea; 2 Qunova Computing, Incorporated, 193 Munji-ro, Yuseong-gu, Daejeon 34051, Republic of Korea

**Keywords:** quantum machine learning, quantum graph neural networks, materials search

## Abstract

Inspired by classical graph neural networks, we discuss a novel quantum graph neural network (QGNN) model to predict the chemical and physical properties of molecules and materials. QGNNs were investigated to predict the energy gap between the highest occupied and lowest unoccupied molecular orbitals of small organic molecules. The models utilize the equivariantly diagonalizable unitary quantum graph circuit (EDU-QGC) framework to allow discrete link features and minimize quantum circuit embedding. The results show QGNNs can achieve lower test loss compared to classical models if a similar number of trainable variables are used, and converge faster in training. This paper also provides a review of classical graph neural network models for materials research and various QGNNs.

## 1. Introduction

The idea to use quantum computers to model physical systems was one of the driving forces of the early development of quantum computers. A prominent quote that enlightens this point comes from the physicist Richard Feynman [1]:

… nature isn’t classical, dammit, and if you want to make a simulation of nature, you’d better make it quantum mechanical.

This is still conjectured until now, as many quantum algorithms for quantum simulation are actively being developed and optimized [2,3]. There are quantum algorithms for calculating ground states, excited states, and the time evolution of molecules and crystals. These algorithms can be divided into pure quantum algorithms such as quantum phase estimation [4] and classical–quantum hybrid algorithms such as the variational quantum eigensolver [5].

Classical machine learning including deep learning has been recently used for predicting the properties of molecules and materials [6,7]. These studies often used datasets of ab initio calculation results as training data to train the models. The trained models could then be used to perform a quick initial screening of materials to find candidates with desired properties. Currently, one of the promising frameworks used for this task is graph neural networks, since structural information can be represented in the form of graph data.

On the other hand, molecule and crystal property prediction by machine learning is still an under-explored research topic in quantum computing. There have been many theoretical studies investigating the possible advantages that quantum machine learning could have over classical machine learning [8,9,10,11,12,13,14], and hence, using quantum machine learning for material design can be an interesting approach.

This paper first reviews classical graph neural network methodologies proposed for the property prediction of molecules and solids. Next, brief introductions to variational quantum machine learning, current quantum graph neural network (QGNN) models, and their applications are presented. Lastly, QGNN models based on the equivariantly diagonalizable unitary quantum graph circuit (EDU-QGC) framework [15] are compared with classical and classical–quantum hybrid models. The novelty of this paper can be found in the QGNN consisting of EDU-QGCs that models atomic bonds in a molecule as discrete link features using a specific parameterization method and EDU ordering, which is used for molecular property prediction.

## 2. Related Works

### 2.1. Classical Graph Neural Networks for Materials Research

A graph is a mathematical structure that consists of nodes (or vertices) and links (or edges), where a link represents the connection between a pair of nodes. While there are many different types of machine learning problems on graphs, in chemistry and materials applications, the most-common task is the regression of graph-wide properties or the classification of graphs. These tasks require that the model output the same value regardless of the order in which the links or the nodes are taken in the graph network model description. In the case where the 3D structure of the graph is given, various symmetries such as rotational or translational symmetries can be inherent in the problem. Building these inductive biases into the machine learning model is an important factor in determining the model’s performance. Graph neural networks are neural networks built specifically for processing graph data.

In order to discuss the details of the models, the message-passing neural network (MPNN) framework will be introduced [16]. The MPNN framework encompasses most models discussed in this section and is even relevant in understanding some quantum models. MPNNs assume an input graph *G* with nodes *N*, links *E*, and optional features of these nodes $\left\{{\vec{x}}_{v}\right\}$ and links $\left\{{\vec{e}}_{vw}\right\}$. There are two phases in the forward pass: the message-passing phase and the readout phase. In the message-passing phase, the hidden feature vector of each node $h_{v}^{t}$ is updated using message $m_{v}^{t+1}$, as written in Equation (1). The message is calculated by aggregating individual message function values from neighboring nodes (Equation (2)). This aggregation (aggr) is invariant about the permutation of nodes, such as the summation of all the messages or taking the maximum.

(1) $$h_{v}^{t+1}=U_{t}\left(h_{v}^{t},m_{v}^{t+1}\right)$$

(2) $$m_{v}^{t+1}=\underset{w\in N(v)}{\mathrm{aggr}}M_{t}\left(h_{v}^{t},h_{w}^{t},e_{vw}\right)$$

After *T* times of message passing, the final node features are passed to a readout function, which calculates the outputs in a node-permutation-invariant manner:

(3) $$y=R(\left\{h_{v}^{T}|v\in N\right\}).$$

**Figure 1:**
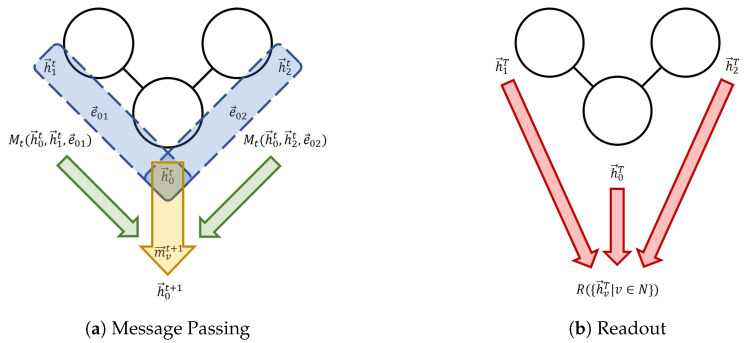
The message-passing neural network (MPNN) framework. (**a**) One message passing step for Node 0. The blue section indicates the process of the calculation of individual messages; the green arrows indicate the aggregation; the yellow arrow represents the node feature update. (**b**) The readout phase.

The updating functions $\left\{U_{t}\right\}$, message calculation functions $\left\{M_{t}\right\}$, and the readout function *R* can be trained. A diagram summarizing the MPNN is shown in Figure 1.

In the case of molecules, it is intuitive to represent them as atomistic graphs (atoms as nodes and bonds as links). For instance, Gilmer et al. (2017) [16] used an MPNN with gated graph-neural-network (GGNN)-type message passing [17] and a set-to-set [18] readout function for various property regression tasks on atomistic graphs. Several variants of this model exist, such as a directed MPNN (2019) [19] model, which uses message passing of directional link features.

Some models use the 3D positional information of each atom for predicting some quantum properties of molecules. SchNet (2018) [20] takes the 3D positions and nuclear charges of each atom in a molecule as the input. The atomic features are updated using filters determined by distances between other atoms. The filter-generating network is also trainable. Recent models have begun to take angle and torsion into account. DimeNet (2020) [21] uses an aggregation of directional embeddings to represent atoms. The update function for the directional embeddings used a radial basis function representation of the interatomic distance and a 2D representation of the angle. The ALIGNN (2021) [22] uses an atomistic graph (atoms as nodes and bonds as links) along with an atomistic line graph (bonds as links and pairs of bonds as links) input. Graph convolution was applied on each graph in an alternating fashion. SphereNet (2022) [23] uses update functions that explicitly take the torsion and angle information as the input. Note that many of these models were used for molecular dynamics prediction and crystal property prediction.

In the case of crystals, representing the input material as a graph is a nontrivial task because of the periodic structure. The CGCNN (2018) [24] uses an undirected multigraph to represent the crystal. Nodes are atoms, and links are determined by a threshold distance and whether or not two atoms share a Voronoi face. This model was used for the prediction of eight properties of crystals and was able to demonstrate potential uses in materials design by observing the characteristics of different chemical environments. This method of representing crystals as a graph was improved in the following studies. MEGNet (2019) [25] aims to provide a unified framework for both molecules and crystals. It added a global feature to the MPNN framework along with node, link, and global feature updates. Crystal graphs were created using connectivity determined by a simple threshold distance. MEGNet was able to outperform SchNet and the CGCNN on the formation energy, band gap, shear modulus, and bulk modulus prediction tasks. The iCGCNN (2020) [26] uses Voronoi neighbors for determining the links and added explicit three-body interactions and link feature updates in the message passing. It outperformed the CGCNN in predicting the stability of inorganic materials and in discovering $TrCr_{2}Si_{2}$-type compounds. Schmidt et al. (2021) [27] used a form of graph attention network [28] on crystal graphs where the connectivity is determined by the five nearest neighbors. This was used to predict the distance to the convex hull on a custom dataset and was used to predict stable quaternary perovskites. The GeoCGNN (2021) [29] uses the 12 nearest atoms within an 8 Angstrom radius as the connection and creates a directed multigraph with directional information encoded into the link features. This allowed for the GeoCGNN to incorporate 3D structural information into the model and outperform the CGCNN, MEGNet, and iCGCNN in predicting formation energy per atom and band gap.

Other structures than molecules and crystals are also studied with graph neural networks. The effective magnetostriction of polycrystalline materials was predicted using graph neural networks [30]. By training on crystals with vacancies or grain boundaries and their atomic properties, stress fields and the energy distribution were predicted for unseen crystals [31]. Polymer property prediction is also an application of graph neural networks [32,33,34]. The classification of amorphous materials into either liquid or glass is also possible [35].

Generative models that output molecules and materials with desired properties can be an alternative to screening. Generative adversarial networks (GANs) [36] and variational autoencoders (VAEs) [37] are the most-widely studied models for chemistry and materials applications. The MolGAN (2018) [38] is a GAN with a generator that creates the adjacency matrix and node features for a molecular graph, a discriminator, which learns to classify whether or not a molecule is generated or not, and a reward network, which steers the model to create molecules with certain properties such as solubility in water. The discriminator and reward networks are graph neural networks. The CGVAE (2018) [39] uses an iterative method to generate molecular graphs. Node features are updated using a graph neural network, while links and their parameters are added by sampling from a learnable distribution, which takes the features of two nodes, their graph distance, initial node features, and node features at that iteration.

**Figure 2:**
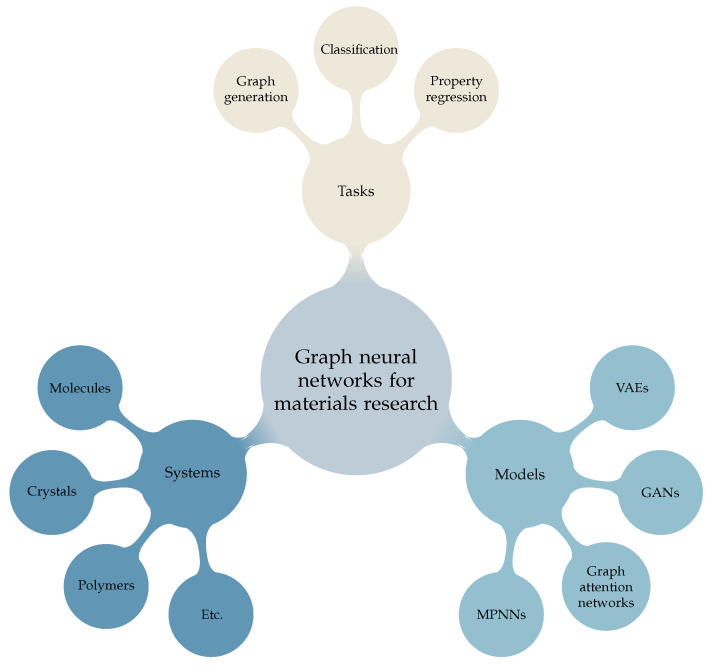
Overview of different systems, models, and tasks discussed in this work for graph neural network methodologies in materials research.

The various problems and models discussed in this subsection are summarized in Figure 2. For more information, the authors suggest a recent review article by Reiser et al. (2022) [6] and another review by Choudhary et al. (2022) [7].

### 2.2. Variational Quantum Machine Learning

Quantum machine learning is the study of applying quantum computing to machine learning problems. There are many different groups of algorithms in this field such as quantum basic linear algebra subroutine-based algorithms [40,41,42,43] and quantum kernel estimation [44,45], but this section will focus on variational quantum machine learning [44,45,46], which is suitable for the near and midterm future applications with noisy intermediate-scale quantum (NISQ) computers [47]. This can be seen as a quantum analog to deep learning, and it is among some of the most NISQ-friendly algorithms.

**Figure 3:**
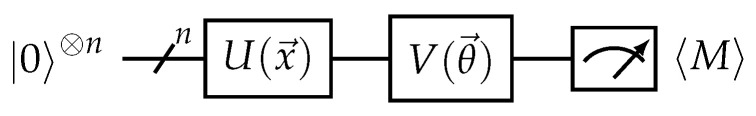
Quantum variational model circuit. *U* is a quantum encoding circuit that maps the input data into a quantum state, while *V* is the quantum neural network (QNN) that can be trained. The model output is the expectation value of an observable *M*.

Variational quantum algorithms are hybrid algorithms where a quantum computer and a classical computer work together in an iterative fashion to solve a problem [48]. A quantum computer performs calculations with initial state preparation, time evolution, and measurement. The quantum computer can execute a parameterized quantum circuit (PQC) in a way that quantum gates for a quantum algorithm, represented as a quantum circuit diagram, can rotate a quantum state according to a set of given classical parameters. Such a PQC can map classical input data samples to output predictions or loss function values. The parts of the circuit that are trainable are often called the quantum neural network (QNN), while the sections that take data inputs are called the quantum encoding circuit. A classical computer determines how to update the parameter values according to the outputs and an optimization algorithm. This feedback loop is repeated until the objective function value is optimized. In the case of machine learning applications, the quantum circuit acts like a parameterized model. A circuit diagram showing an example quantum circuit for a variational QGNN is given in Figure 3.

**Figure 4:**
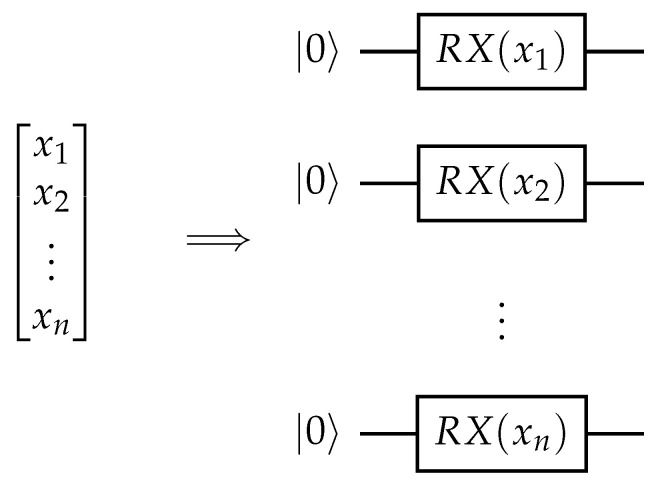
An example of quantum encoding. Individual elements of an input classical vector are used as the rotation angles of rotation Pauli X (RX) gates, creating a quantum state.

When working with classical data, it is important to be able to efficiently represent the input as a quantum state. A key idea in variational quantum machine learning that allows this is quantum encoding. The classical data are input as rotation angles in quantum gates to prepare a state that represents the data. A diagram summarizing this concept is shown in Figure 4. This scheme allows for the Hilbert space to be used as a feature space. Interestingly, the quantum encoding method affects the types of functions the quantum machine learning model can learn [13]. Repeating the encoding circuit throughout the variational model is called re-uploading and can help increase the expressibility of the model [13]. It has been also shown that one could construct a learning problem where one would be able to solve the task with one qubit in the re-uploading case, while a model without re-uploading would require a number of qubits affected by the input data dimension [49]. Some works even focused on framing supervised learning as training the encoding and using an optimal measurement for separating the two ensembles of states [50].

**Figure 5:**
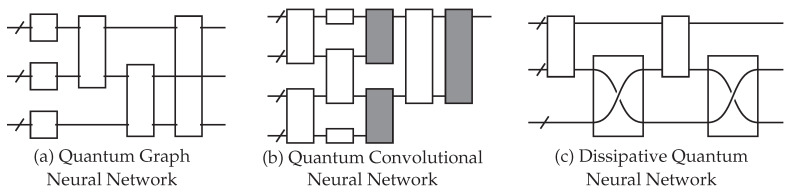
Examples of different QNN architecture designs. Each empty gate has trainable parameters. (**a**) The quantum graph neural network (QGNN) is used for processing graph-structured data. The circuit structure is dependent on the input graph. (**b**) The quantum convolutional neural network has a convolution operator (white) and a pooling operator (gray). (**c**) The dissipative quantum neural network represents each neuron as a group of qubits, and unitary operators transform one layer to another. This example is an input layer of one perceptron (the top group of qubits) being mapped to a layer of two perceptrons (the bottom two groups of qubits).

Just like classical neural networks, QNNs have different architecture designs depending on the structure of the gates. Some examples include the quantum convolutional neural network [51], the quantum graph neural network [15,52,53,54], and the dissipative quantum neural network [55]. These architecture designs are explained further in Figure 5. The models are also divided by the task that they are designed for. There are quantum autoencoders [56], quantum classifiers [44], quantum generative adversarial networks [57,58], and so on.

Training variational QNNs is often limited by the “barren plateau” phenomenon [59]. Under certain conditions, the training landscape of the variational parameters becomes flat, which is characterized by an exponentially vanishing variance of the gradient. Unlike classical neural networks where the gradient vanishes as the number of layers grows larger [60], QNNs face this problem in general when the number of qubits increases. This prohibits the training of quantum models designed for high-dimensional data, even when gradient-free optimization algorithms are used [61]. Barren plateaus are affected by quantum noise [62], as well as the design of the cost function [63]. It is also dependent on the quantum circuit architecture: for example, the quantum convolutional neural network [64] and permutation equivariant quantum circuits [65] do not suffer from barren plateaus, while dissipative quantum neural networks show barren plateaus [66]. Different training and initialization methods have been suggested to solve this problem [67,68,69].

An interesting variant of QNNs is the hybrid classical–quantum neural network [54,70,71,72,73,74]. Since one can calculate gradients for the QNN layers [75,76,77], it is possible to create a neural network with classical and quantum layers. Quantum encoding is used to turn classical data into quantum states, and measurement is used to turn quantum states into classical data. This scheme is expected to improve QNNs. For instance, it is often difficult for QNNs to work with high-dimensional data since NISQ computers are limited in their qubit numbers. Using a hybrid classical–quantum neural network can help alleviate this problem by allowing the classical neural network to reduce the data dimension for the QNN layers.

Whether or not variational quantum machine learning using NISQ machines will show useful advantages over classical machine learning and how this advantage will be quantified are still open questions. Nevertheless, there have been many studies aiming to find conditions and situations where quantum machine learning is superior to classical machine learning.

Sample complexity is a candidate for quantum advantage. When trying to learn a unitary in a supervised manner, one may be able to surpass the classical no-free-lunch theorem by using entangled input states [8]. In some tasks for learning properties or outcomes of quantum states and processes, a model that has access to a quantum memory may be able to achieve low prediction error with exponentially fewer copies/access of the state/process compared to classical models [9,10].

The generalization error of QNNs is determined by the training set size and number of trainable parameters. Therefore, a task where a QNN with a relatively small number of trainable parameters and a classical model achieve similar training error may show quantum advantage [11].

Quantum neural networks were shown to be expressive and trained with fewer iterations compared to classical neural networks [12]. In fact, it can become a universal function approximator if the encoding allows for a wide spectrum [13]. PQCs as generative models also possessed better expressivity compared to classical models [14].

### 2.3. Quantum Graph Neural Networks

There have been several proposals for quantum variational machine learning models for graph-structured data. Some of these works are summarized below in chronological order.

Verdon et al. (2019) [52] proposed a QGNN whose rotation-generating Hamiltonian operators have the topology of the problem graph. This means that the state of each node is represented in a Hilbert space, and there are node-local terms along with interactions between nodes connected with links. More specifically, for an input graph with nodes V and links E, a single layer of this QGNN would be made up of operators that can be written as Equation (4).

(4) $$e^{-i\hat{H}\left(\theta ,\phi \right)},\hat{H}\left(\theta ,\phi \right)=\sum_{(j,k)\in E}\sum_{r\in I_{jk}}\theta_{rjk}{\hat{O}}_{j}^{r}⊗{\hat{P}}_{k}^{r}+\sum_{v\in V}\sum_{r\in J_{v}}\phi_{rv}{\hat{R}}_{j}^{v}$$

$\hat{O},\hat{P},\hat{R}$ are operators acting on the node designated in the subscript, while θ and ϕ are trainable variables. $I_{jk}\left(J_{v}\right)$ are index sets of the corresponding link (nodes), which allows for multiple Hamiltonian terms per link (node). Depending on the coefficients of the Hamiltonian terms, models with various properties can be created such as the quantum graph recurrent neural network and the quantum graph convolutional neural network. These models were used for various toy problems that included Hamiltonian dynamics learning and graph clustering.

Beer et al. (2021) [53] first defined graph-structured quantum data. Given a graph, each node corresponds to a quantum state, and there is a link between two quantum states if they are within a certain information theoretical distance of each other. Then, one can define a supervised learning scenario where each node should be mapped to a certain labeled quantum state. This work provides loss function designs and training methods for this task using dissipative quantum neural networks.

Zheng et al. (2021) [78] created a model with state preparation, quantum graph convolution, quantum pooling, and quantum measurements. During state preparation, the node features are encoded into a quantum register per node, and the connectivity is encoded as |0〉 or |1〉 on the node-pair-representing qubits. The convolution is then represented as controlled two-node unitaries and the pooling as measurement-conditioned unitary operations on the node qubits. The remaining qubits are measured for the model output value. This was used for the classification of handwritten digits.

Tüysüz et al. (2021) [54] proposed a hybrid classical–quantum graph neural network. Here, the link and node features of an input graph are updated using link and node networks like a classical graph neural network. However, in this model, a QNN layer is used in between classical fully connected layers to create the link and node networks. This model was applied to the task of particle track reconstruction at the Large Hadron Collider and showed similar results with classical models.

**Figure 6:**
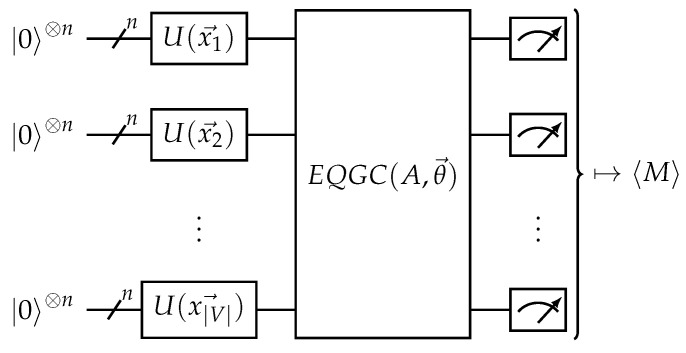
The equivariant quantum graph circuit (EQGC) framework. Given an input graph with nodes *V*, adjacency matrix *A*, and node features ${\left\{{\vec{x}}_{i}\right\}}_{i\in V}$, the circuit drawn above is used. The EQGC is equivariant on the permutation of nodes and is trainable, while the measurement is invariant on the permutation of nodes.

Mernyei et al. (2022) [15] used the concept of equivariance taken from geometric machine learning to create equivariant quantum graph circuits (EQGCs). Given input graph data made up of a tuple of nodes, links, and node features, some fixed number of qubits are assigned to each node. Next, the node features are encoded into quantum states by applying a parameterized unitary onto the corresponding qubits. Next, a node-permutation-equivariant quantum circuit is applied, followed by a node-permutation-invariant measurement. Equivariance to permutation means that the permutation can commute with the quantum circuit. The node-permutation-invariant measurement is simple to design, such as the average of the expectation values of a node-local observable over all nodes. This framework can be visualized as the circuit in Figure 6.

There are two main ways of constructing an EQGC suggested in the original paper. One is the equivariant Hamiltonian quantum graph circuit (EHQGC), where the QNN is made up of unitaries with rotation-generating Hamiltonian operators that have the same topology as the input graph. Note that this is similar to Verdon et al.’s proposition for a QGNN.

Another is the equivariantly diagonalizable unitary quantum graph circuit (EDU-QGC). EDU-QGCs are made up of node layers and link layers. Each node layer is made up of a node-local unitary operator that acts on all the nodes. Each link layer is made up of equivariantly diagonalizable unitaries (EDUs) acting between two nodes connected by a link. An EDU is defined as a unitary acting on two nodes that can be decomposed in the form of Equation (5).

(5) $$\mathrm{EDU}=\left(V^{†}⊗V^{†}\right)D\left(V⊗V\right)$$

**Figure 7:**
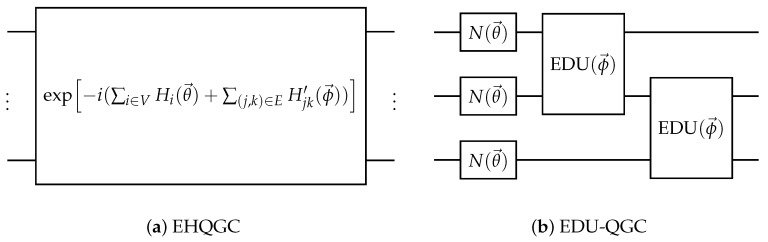
Examples of two different EQGC implementation methods. (**a**) is an example of the equivariant Hamiltonian quantum graph circuit (EHQGC), and (**b**) is an example of the equivariantly diagonalizable unitary quantum graph circuit (EDU-QGC).

Here, the unitary operator *V* acts on one node, while *D* is a diagonal unitary that acts on two nodes. Since the EDU commutes with the SWAP operator and a copy of itself acting on other qubits, the link layer is equivariant on the permutation of the nodes. Note that EDU-QGCs can be represented as EHQGCs. There are also some expressibility results about EDU-QGCs. EDU-QGCs can approximate any real-valued function over bounded graphs and can pass the 1-WL test, which deterministic classical MPNNs cannot pass [79]. Examples of the two EQGC methodologies are given in Figure 7.

## 3. Methods

### 3.1. Data Preprocessing Methods

**Figure 8:**
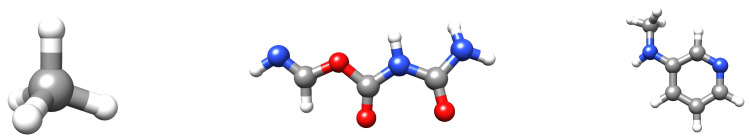
Examples of molecules in the QM9 dataset. The white atoms are hydrogen; the grey atoms are carbon; the red atoms are oxygen; the blue atoms are nitrogen. Visualization was achieved with UCSF Chimera [83].

The QM9 dataset was used to train the QGNN [80,81]. This dataset contains organic molecules with up to 9 non-hydrogen atoms, and their properties were calculated using DFT. The molecules included in the dataset ranged from the simplest being methane to more complicated molecules with substructures such as aromatic rings, chain-like structures, and other 3D structures. Some examples are shown in Figure 8. A simplified version of the preprocessing method from Gilmer et al. [16] was used to turn these raw data into graph-structured data. To summarize the preprocessing method, all non-hydrogen atoms were chosen to be nodes, and the bonds between them were the links. Table 1 shows the features that were extracted. Extraction was performed by using RDKit [82] to analyze the given SMILES representation of the molecule. Note that this resulted in 3D structural information being lost. However, there are two reasons why this method was chosen. First, the purpose of this experiment was to create and iterate upon a simple working model. Second, when given a molecule with which to perform inference, the 3D structural information may not be known beforehand. This is because often determining the 3D structure of a molecule is a difficult task in itself.

One method for enhancing the molecular graph is adding a master node. The master node does not represent an atom, but is connected to all other nodes. The node and link features related to the master node are different from all other node or link features. In the QGNN, this translates into a unique initial quantum state for the master node and separate trainable parameters for the node operator and EDUs. This preprocessing method was used by Gilmer et al. [16] to model long-range interactions between atoms.

### 3.2. Quantum Graph Network Model Design

The design of the EDU-QGC model is divided into 3 sections: the quantum encoding of node features, the EDU-QGC, and the readout function. The various design choices for each section are explained in the following paragraphs. In the text, an RP gate, where P is a Pauli word, is a unitary gate with a single parameter θ and is expressed as exp{−iθP/2}.

#### 3.2.1. Quantum Encoding

One qubit was assigned to each node, and encoding was performed with an RY gate followed by an RZ gate. The initial qubit state was determined by the node feature values. Three different encoding methods with three different input features, detailed in Table 2, were tested in this experiment. For the case of the (Atomic Number) encoding, the 4 possible qubit states were separated as far as possible from each other, resulting in them being the vertices of a tetrahedron inscribed in the Bloch sphere. For the (Atomic Number and Number of Hydrogens) encoding, each feature was assigned to either an RY or RZ gate, and the possible angles were evenly spaced in [0,2π]. This same methodology was used for the (Atomic Number, Aromaticity, and Hybridization) encoding, except that two features (Aromaticity and Hybridization) were used to determine the RZ rotation angle.

#### 3.2.2. EDU-QGC

**Figure 9:**
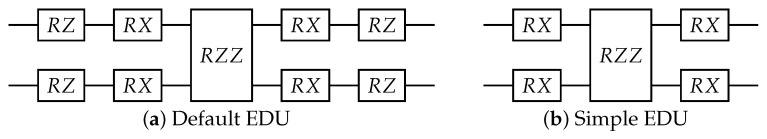
The equivariantly diagonalizable unitary (EDU) circuit design. The single qubit gates are parameterized with trainable link variables, while the diagonal two-qubit gate is parameterized with a trainable parameter dependent on the layer. (**a**) The default EDU was used throughout the experiment. If not otherwise specified, this is the EDU that was used. (**b**) A simpler EDU was used to test the effect of the expressibility of the EDU on the model performance.

The EDU-QGC design needs to be different from the original suggested architecture in order to process link features. Since EDUs represent links, the EDU was chosen to be parameterized by trainable variables that represent a possible link feature. To be more specific, the diagonal unitary in the EDU was parameterized by a trainable variable that was different for each layer, while the node-local unitary in the EDU was parameterized by trainable variables that were determined by the bond order. Thus, even if the bond order was different, the EDUs inside the same layer would share the diagonal unitary’s rotation angle while the node-local unitaries would be different. The EDU architectures used in this experiment are shown in Figure 9. The node-local unitary used in the node layer was the arbitrary single-qubit unitary with 3 trainable parameters in the case of the default EDU and the RY gate in the case of the simple EDU. RZZ was used as the diagonal unitary since the input graph was an undirected graph. Thus, it is natural to use a gate that is unaffected when the two connected nodes are swapped.

**Figure 10:**
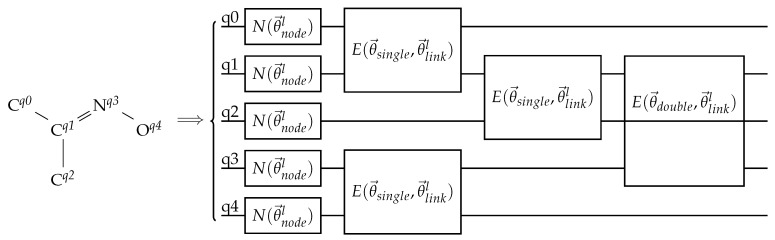
An example of a bond-information-encoding EDU-QGC layer. The input molecule is drawn on the left without its hydrogen atoms. The superscripts are the assigned qubit indices. The circuit on the right shows one EDU-QGC layer. *N* and *E* are node-local unitaries and EDUs, respectively. ${\vec{\theta }}_{node}^{l}$ and ${\vec{\theta }}_{link}^{l}$ are trainable parameters that depend on the layer number *l*, while ${\vec{\theta }}_{single}$ and ${\vec{\theta }}_{double}$ are trainable variables that depend on the bond order. Note that the single bonds are applied first, then the double bond.

A naive use of this parameterization scheme results in a breaking of the node permutation equivariance. This is because EDUs with different parameter values will generally not commute with each other. In order to solve this issue, an ordering of the EDUs was enforced. The EDUs representing the same link feature would be applied together, while the order of the link features would be fixed. In this experiment, the following order of the bonds was enforced: single bond → aromatic bond → double bond → triple bond. An example of an EDU-QGC with bond information is shown in Figure 10.

This experiment used two different types of variational circuits. One has simple repetitions of the EDU-QGC layer, while the other uses a re-uploading model [13]. When there is no explicit mention of re-uploading, the QGNN will use a simple repetition of the circuit architecture. Re-uploading repeats the quantum encoding after every EDU-QGC layer. This has been shown theoretically to improve the expressibility of the model.

#### 3.2.3. Readout Function

The readout function is a measurement that is invariant about the permutation of the qubits. Two readout functions were tested in this experiment. The readout function given in Equation (6) is called the local readout function, as it is made up of node-local terms.

(6) $$r_{0}+\frac{r_{1}}{\left|V\right|}\sum_{v\in V}\langle Z_{v}\rangle$$

The readout function represented as Equation (7) is called the global readout function, since there is a term that acts on all nodes.

(7) $$r_{0}+\frac{r_{1}}{\left|V\right|}\sum_{v\in V}\langle Z_{v}\rangle +r_{2}\langle \underset{v\in V}{⨂}Z_{v}\rangle$$

The $Z_{i}$ operators are the Pauli Z operators acting on qubit *i*, and the *r* parameters are all trainable variables. The *r* variables are needed since the expectation values are all bounded between −1 and 1.

### 3.3. Neural-Network-Assisted Quantum Encoding

**Figure 11:**
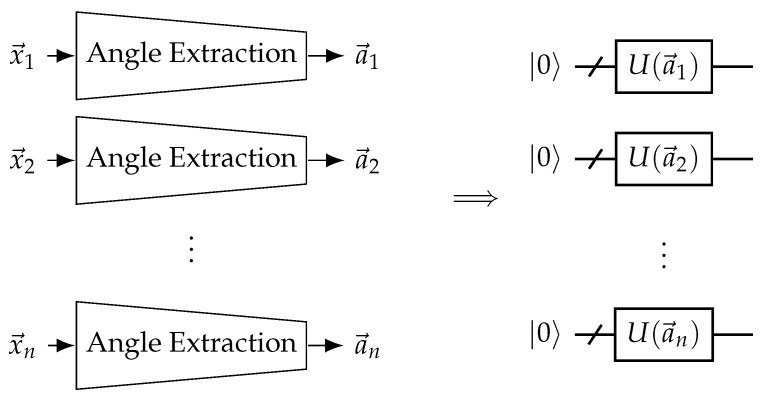
Neural network-assisted quantum encoding EQGCs. Explicit input features $\left\{\vec{x}\right\}$ are transformed into rotation angles $\left\{\vec{a}\right\}$ for the QGNN to use for encoding using a trainable angle extraction neural network. In the case of EQGCs, the same angle extraction network should be used for all nodes.

The quantum encoding methods used in typical QNNs are quite empirical and arbitrary. We aimed to automate the process of finding the optimal encoding method by forming a hybrid classical–quantum neural network where the classical neural network only aids in the encoding process. Explicit node features are input into an artificial neural network, which outputs rotation angles that are to be used in the quantum encoding step. This artificial neural network will be called the angle extraction network and is trainable. We call this model the neural-network-assisted quantum encoding EQGC. A diagram summarizing this idea is shown in Figure 11. Note that similar ideas were used in previous works, especially in the context of classical–quantum transfer learning [70] and quantum few-shot encoding learning [74].

In order to directly compare with the fixed encoding methods detailed in Table 2, the (Atomic Number and Number of Hydrogens) input case and the (Atomic Number, Aromaticity, and Hybridization) input case were tested. Since the features were discrete, one-hot encoding was used on the features. The angle extraction networks had an output layer of dimension 2 and one hidden layer of dimension 4. All layers were fully connected. The activation function between the input and hidden layer was ReLU, and the activation function between the hidden layer and output layer was 2π×Sigmoid. This was to ensure that the output was between 0 and 2π. One output element was input into an RY gate and the other into the following RZ gate.

### 3.4. Classical Graph Neural Network Models

The training results of the QGNNs should be compared to classical graph neural network models. However, the current state-of-the-art classical models use different preprocessing methods, as well as more complicated models with more trainable parameters. Therefore, in order to isolate the effect of introducing QGNNs to this problem, some custom classical models were created.

The following conditions were imposed on the classical model. The classical model was set to have 3 message-passing steps, as the QGNN used up to 3 EDU-QGC layers. The node feature dimension was also kept to 2 since the quantum network assigned one qubit to each node. The readout function was the mean of the final layer’s node feature vectors since the quantum network also used a simple mean.

Two different classical graph neural network layers were used. One was a GGNN-type convolutional layer [17], which was used on the QM9 dataset with similar preprocessing methods [16] and in point cloud classification [84]. The update of the node features ${\vec{x}}_{i}$ was performed with Equation (8).

(8) $${\vec{x}}_{i}^{t+1}=\Phi {\vec{x}}_{i}^{t}+\underset{j\in N(i)}{\mathrm{aggr}}NN\left({\vec{e}}_{ij}\right){\vec{x}}_{j}^{t}$$

NN is a neural network that outputs a matrix when the link feature is input. In the experiment, this network always has one hidden layer of dimension 2. aggr is an aggregation function, and N(i) is the neighbor of the node *i*. The addition, mean, and maximum functions were tested for the aggregation function. Another graph neural network layer was the convolutional layer of a graph convolutional network (GCN) [85], which was chosen for its simplicity. The link features were used as entries in the input adjacency, matrix and ReLU was used for the activation between GCN layers. Various models were created by combining these layers.

### 3.5. Training and Evaluation Methods

The entire dataset was randomly reduced to 10,000 training samples, 1000 validation samples, and 1000 testing samples due to simulation time concerns. The quantum circuits were simulated using conventional server-class computers. While this is not more efficient than using quantum hardware, it eliminated the effect of quantum noise and finite shot error, which will help compare the ideal performance of the various models. All quantum circuit simulation was achieved with Pennylane [86], and PyTorch [87] was used for the classical neural network calculations. The classical graph neural networks were calculated using PyTorch Geometric [88]. During training, a batch size of 128 was used.

Learning was achieved with an Adam optimizer [89] with learning rate 0.01 and $\beta_{1}=0.9$, $\beta_{2}=0.999$. The loss function was the mean-squared error between the model output and the target value. While the dataset contained many targets, this experiment focused only on the highest occupied molecular orbital—lowest unoccupied molecular orbital (HOMO-LUMO) energy gap. The validation loss was calculated every 10 epochs for 150 epochs, and the weights with the lowest validation loss were chosen. In the case of the classical models, 3 different training runs with different initial parameter values were used to determine the optimal weights. For the quantum or classical–quantum hybrid models, only one run was used, and the initial quantum weights were all initialized to 1.

## 4. Results

### 4.1. Pure Quantum Graph Neural Network Model Training Results

#### 4.1.1. Quantum Encoding Method Comparison

**Figure 12:**
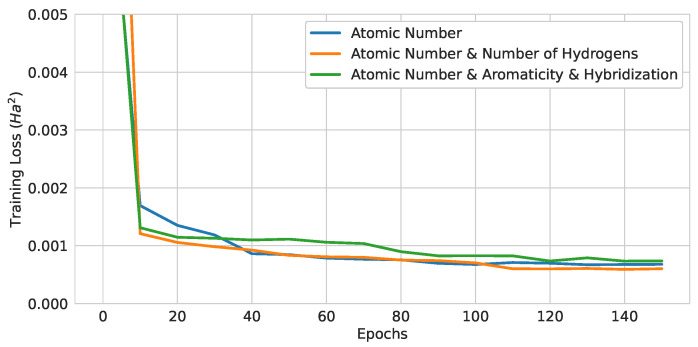
Training curves of 3-layer pure QGNNs with different encoding strategies.

The training curves of three different QGNNs are shown in Figure 12, and the test loss values of trained models are summarized in Table 3. The models only differed in the encoding methods, while the rest of the model used three layers of the EDU-QGC and the local readout function. Therefore, the number of quantum gates in these models were the same, and the quantum computational cost was the same. Since the model with the (Atomic Number and Number of Hydrogens) encoding achieved the lowest test loss, this was used as the baseline model.

#### 4.1.2. Number of Layers Comparison

**Figure 13:**
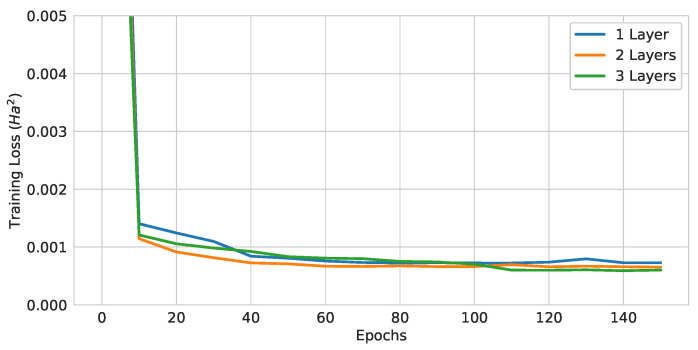
Training curves of pure QGNNs with different numbers of layers.

QGNNs with (Atomic Number and Number of Hydrogens) encoding and local readout functions were trained with different numbers of layers. The quantum computational cost increased proportionally with the number of layers. The resulting test losses are shown in Table 4 and the training curves in Figure 13. As expected, the model improved with more layers. This effect was expected to saturate, as the expressibility of QGNNs saturated as the number of layers increased [90].

#### 4.1.3. Readout Function Comparison

**Figure 14:**
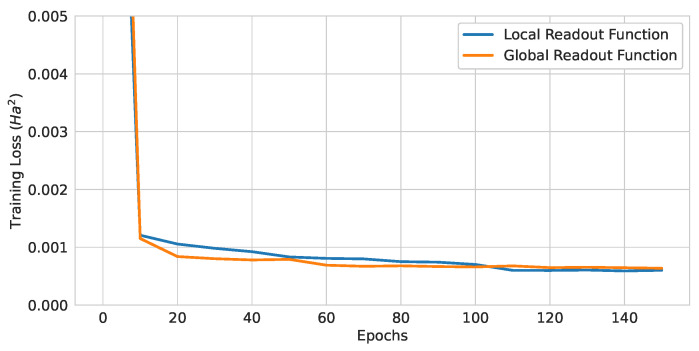
Training curves of QGNNs with different readout functions.

In order to test the effectiveness of the readout functions, two models with (Atomic Number and Number of Hydrogens) encoding and three layers were trained with different readout functions. The number of quantum gates was the same for the two models. The test losses are summarized in Table 5 and the training curve in Figure 14. Note that the local readout function is a special case of the global readout function ($r_{2}=0$). Therefore, one could assume that the global readout results should be at least as good as the local readout results. However, the local readout outperformed the global version. One possible explanation for this result is that the optimization algorithm is prone to be caught in the barren plateau problem with the global readout function [63].

#### 4.1.4. EDU-QGC Architecture Comparison

**Figure 15:**
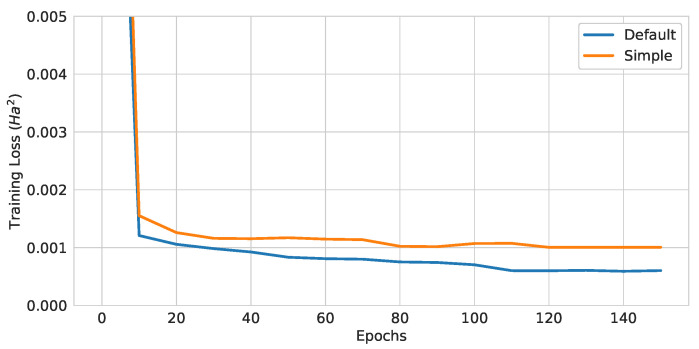
Training curves of QGNNs with different EDU-QGC architectures.

Three layers of the default EDU-QGC and the simple EDU-QGC were trained with local readout and (Atomic Number and Number of Hydrogens) encoding. Note that the additional one-qubit operations in the default EDU-QGC can be performed in parallel and the RZ gates between adjacent bond orders can be combined into a single gate. Therefore, the default EDU-QGC had 2fl more single-qubit gate layers than the simple EDU-QGC, where *f* is the number of different edge features in the input graph and *l* is the number of EDU-QGC layers. The training curves are plotted in Figure 15, and the test losses are summarized in Table 6. The default EDU-QGC had EDUs and node layers that had higher expressibility compared to the simple EDU-QGC case. This resulted in a better performance of the default than that of the simple model.

#### 4.1.5. Other Modifications

**Figure 16:**
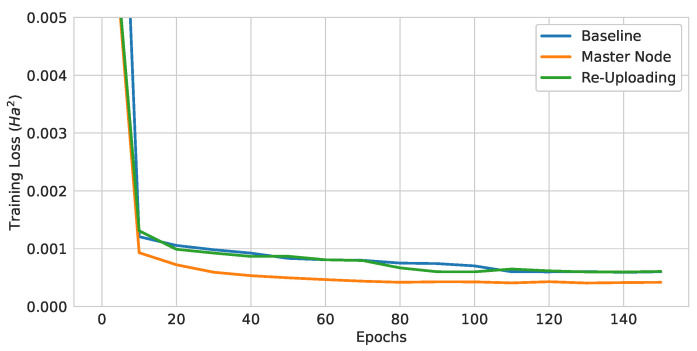
Training curves of QGNNs with different modifications for higher expressibility.

Two modifications were also tested, specifically aiming at increasing the model performance. One was the master node preprocessing, and the other was the re-uploading scheme. The test losses and the training curves are shown in Table 7 and Figure 16, respectively. The impact of the master node showed roughly a 31% improvement over the baseline, which is quite significant. However, this came at the cost of one extra qubit, about twice the number of trainable variables, and about twice the number of two-qubit gates. While the theoretical results showed that the re-uploading model was more powerful than a model with an encoding circuit at the beginning, the test loss was worse than that of the baseline, despite using six more single-qubit gate layers than the baseline model.

### 4.2. Neural-Network-Assisted Quantum Encoding Model Training Results

**Figure 17:**
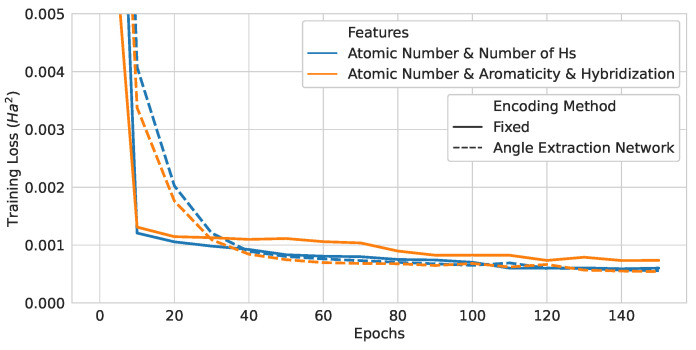
Training curves of neural-network-assisted quantum encoding models and their quantum counterparts.

Neural-network-assisted quantum encoding models with two different node feature inputs were trained. The training curves are drawn in Figure 17, and the test losses are shown in Table 8. For both input features, the angle extraction network introduced a significant performance increase and test loss decrease by 9.6% and 30.6%, respectively. Here, 50 and 46 more training variables were used for the (Atomic Number and Number of Hydrogens) and (Atomic Number, Aromaticity, and Hybridization) encodings, respectively. In terms of quantum computational resources, all of the models used the same number of gates.

### 4.3. Classical Graph Neural Network Model Training Results

**Figure 18:**
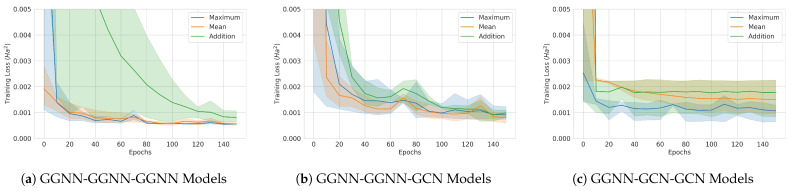
Classical model training curves. The solid lines are averages over 3 runs, and the 95% confidence intervals are also shown. (**a**) GGNN-GGNN-GGNN model. (**b**) GGNN-GGNN-GCN model. (**c**) GGNN-GCN-GCN model.

Three different convolutional layer architecture designs were trained and tested. The training curves are plotted in Figure 18, and the test losses are given in Table 9. Each training curve in solid line is the average over three runs with different initial weights, and the 95% confidence interval is shaded. The large confidence intervals show that the initial weight values seemed to greatly affect the training results of the classical models. Only the weights out of all of the runs with the lowest validation loss were used for testing. Interestingly, the best-performing models had different aggregation functions across the architectures. Overall, the model performance improved with more trainable parameters.

## 5. Discussion

### 5.1. The Proposed Quantum Network Model on Quantum Hardware

The numerical experiments in this study were achieved by simulating the quantum circuits on conventional classical computers. It is an important issue whether or not our scheme is feasible to be executed as quantum circuits on near-term quantum hardware. The novel QGNN models designed in this paper require a number of qubits proportional to the number of nodes per graph. They also require high qubit connectivity, since when the molecular graph is not able to be mapped to the qubit connectivity graph, extra computational resources such as SWAP gates are required to run the circuit. Trapped ion quantum computers have all-to-all connection and high gate fidelities, which we consider to be the best hardware for the quantum network models. Currently, the leading trapped ion quantum computers provide up to 32 physical qubits, such as by IonQ Forte [91]. The effect of noise will be likely the limiting factor rather than the number of qubits when working with the QM9 dataset. Nonetheless, it will be a critical challenge to model a larger molecule by the proposed QGNN considering hardware limitations.

### 5.2. Model Performance Evaluation and Comparison

**Figure 19:**
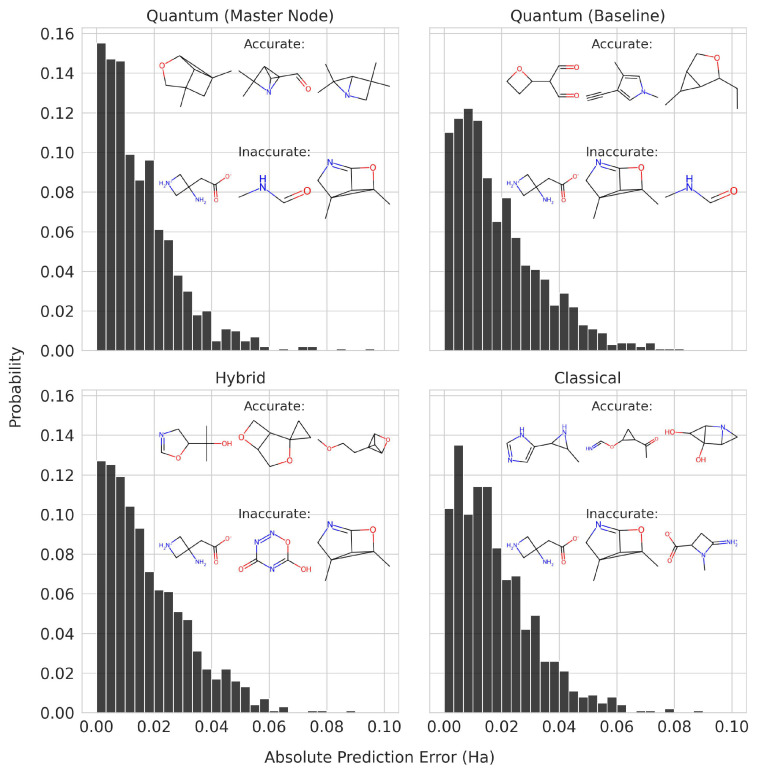
Absolute prediction error distribution of quantum, hybrid, and classical models on the test dataset molecules. The molecules for which the models have accurate and inaccurate predictions are drawn.

The model performance of the best quantum, classical, and hybrid models can be analyzed more in detail. The baseline QGNN, the baseline and master-node QGNN, the (Atomic Number and Number of Hydrogens) hybrid model, and the GGNN-GGNN-GGNN classical model with mean aggregation were chosen for their low test loss. The probability distribution of the absolute prediction error was calculated using the test dataset, as shown in Figure 19. The models with the lower test loss showed a probability distribution more concentrated about 0 error. The three molecules with the most-accurate and most-inaccurate predictions are also drawn. For the most-inaccurate molecules, some were common among the different models. On average, the inaccurate molecules had a HOMO-LUMO gap energy of 0.1900 Ha with a standard deviation of 0.05203 Ha, while the accurate molecules had that of 0.2684 Ha with a standard deviation of 0.04446 Ha. Hence, the models tended to give accurate predictions for molecules with high HOMO-LUMO gaps.

In order to see if aromatic rings in the molecules resulted in a different prediction performance, we calculated the mean absolute error on molecules in the test dataset with and without aromatic rings. The results are shown in Table 10. All models had better predictions on molecules without aromatic rings, but the difference was more pronounced in the pure QGNNs compared to the hybrid or classical models.

Lastly, the hybrid models and QGNNs can be compared to the state-of-the-art classical models using the test mean absolute error values. The ALIGNN (2021) reported a test error of $1.4001\times 10^{-3}\;\mathrm{Ha}$, while SphereNet (2022) was evaluated at $1.1429\times 10^{-3}\;\mathrm{Ha}$. This was about 10-times lower than the best-performing QGNN, which achieved a mean absolute error of $1.5106\times 10^{-2}\;\mathrm{Ha}$. Note that the training and testing methods were different; however, one can gain a general sense of the performance difference in these models. We suggest that the current QGNN is simple and there is room for improvement.

### 5.3. The Effect of Quantum Encoding Methods

The quantum encoding method used in a quantum machine learning model is a key decisive factor in the success or failure of the models. Thus, it was important in this study to test and compare various encoding methods. When comparing the three purely quantum encoding methods, the number of features being used seemed to be important. In the case where three features was encoded into a single pure qubit state, there was an effective dimension reduction. In deep learning models, this feature extraction process is trainable, but in the case of the pure QGNNs, this is fixed. Thus, it is difficult to optimize in the quantum case. In addition, the number of possible encoded quantum states would generally increase when the number of features increases. This means a large overlap in the latent space between two different inputs. On the other hand, when only one feature is used, it seems to not contain enough information about the molecule for the model to make an accurate prediction. Thus, we concluded that, in order to create a pure QGNN that can meaningfully incorporate high-dimensional input features, more qubits should be allocated to each node to represent atoms in a molecule. However, finding an exact optimal number of qubits per node and the corresponding encoding method are open questions and likely dependent on the problem setting.

This study suggested that an additional neural network can be a good candidate to reduce the complexity of the problem of high-dimensional input quantum encoding. The results showed that this scheme, to optimize the encoding process using an angle extraction network, improved the model performance over heuristic angle selection. Another possible encoding scheme for future studies is to treat the EDU-QGC model as a single graph neural network layer. Then, one could use the EDU-QGC model in between several classical graph neural network layers. This would also allow for high-dimensional input features and more complex readout functions.

**Figure 20:**
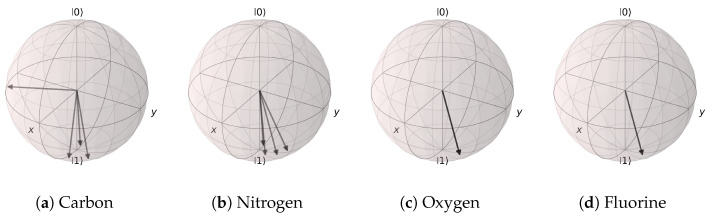
Angle extraction network results for the (Atomic Number, Aromaticity, and Hybridization) model. All possible atomic features are grouped together by the atom type. (**a**) Carbon atom states are mostly distributed near |1〉, except for the non-aromatic $sp^{2}$ hybridization case. (**b**) Nitrogen, (**c**) oxygen, and (**d**) fluorine atoms are all distributed near the |1〉 state.

**Figure 21:**
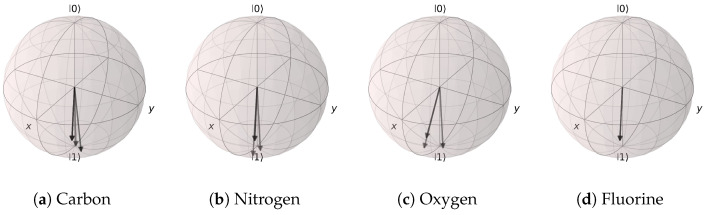
Angle extraction network results for the (Atomic Number and Number of Hydrogens) model. The states are distributed near |1〉. (**a**) carbon atoms, (**b**) nitrogen atoms, (**c**) oxygen atoms, and (**d**) fluorine atoms.

The mapping results of the trained angle extraction networks are represented on the Bloch spheres in Figure 20 and Figure 21. In the case of the (Atomic Number, Aromaticity, and Hybridization) input, most of the states were mapped to states near |1〉, except for the non-aromatic carbon atom with $sp^{2}$ hybridization. Furthermore, all oxygen atoms, fluorine atoms, and aromatic nitrogen atoms with hybridization $sp^{2}$ were mapped to the same quantum state. For the (Atomic Number and Number of Hydrogens) input, all of the states were distributed near |1〉. This is highly counter-intuitive, as the highly localized distributions of the states seem to suggest that the atomic features had little effect in the context of the EDU-QGC model.

In order to test this observation, a three-layer EDU-QGC model with a local readout function was trained, where the encoded state for each atom-representing qubit was |1〉. The optimization and evaluation methods were kept consistent with the main results. This model showed a test loss of $6.4058\times 10^{-4}\;{\mathrm{Ha}}^{2}$, which is an improvement over the fixed encoding of (Atomic Number, Aromaticity, and Hybridization), but performed worse than all neural-network-assisted encoding models or other fixed methods. Therefore, while the visualization of the learned encodings suggests that the states were concentrated about |1〉, the slight differences were meaningful.

### 5.4. Convergence Tendencies in Training

**Figure 22:**
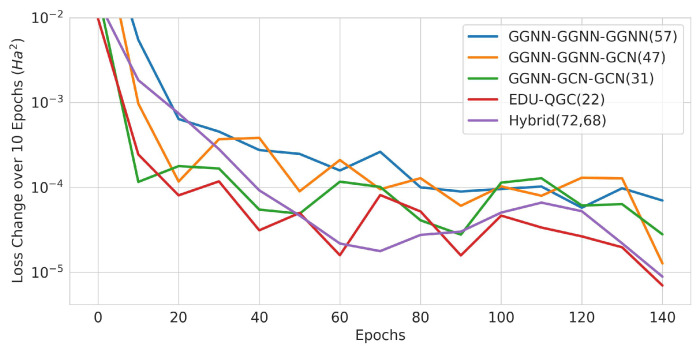
Change in training loss in 10-epoch intervals. The solid lines are averages of the runs, and the x axis is the beginning epoch number of the interval. The number of trainable variables is written in the legend in parenthesis.

The absolute difference in training set loss was calculated in 10 epoch intervals starting from Epoch 0 in order to observe the speed for training convergence. The models with the (Atomic Number and Number of Bonded Hydrogens) inputs and three layers were grouped together depending on their number of trainable variables. The resulting graph in Figure 22 shows that QGNNs have a tendency to converge faster than classical models on average and reached a training loss change of $10^{-5}\;{\mathrm{Ha}}^{2}/10$ epochs the quickest. The classical models showed faster convergence as the number of trainable variables decreased. Interestingly, the hybrid classical–quantum models showed similar convergence with classical models, which have fewer variables.

### 5.5. Computational Cost of the Quantum Graph Neural Network Models

Assessing the computational cost of the QGNN can be divided into three scopes: the quantum circuit structure, calculating the gradient, and the actual training. In terms of the quantum circuit structure, many of the gate operations were able to be performed in parallel on a single quantum processor. The quantum encoding step and the node operator in the EDU-QGC applied unitary operators to each node-representing subsystem, so these unitary operators can be applied at the same time. Thus, there was no scaling of these operators depending on the input graph size. For the case of the EDU-QGC, the number of EDUs will scale linearly with the number of edges. However, EDUs that act on different qubits were able to be applied at the same time. The time cost was then linear to the chromatic index of the input graph, where it is known that a simple graph with maximum degree *n* can be colored with at most n+1 colors (Vizing’s Theorem [92]). Thus, the quantum circuit should have a time cost that is at most linear with the number of nodes. Compared to the MPNN, which has a time cost linear with the number of edges and nodes, this can be an improvement depending on the connectivity of the input graphs. Scaling of the circuit with respect to the node feature dimension or link feature dimension is not consistent. This will depend on the form of quantum encoding and the number of possible link features.

When using quantum hardware, one needs to run the same circuit multiple times to estimate an expectation value. However, a previous work has shown that, even when using few shots for the estimation, one can train a quantum circuit [93]. Thus, we suggest that calculating the gradient is efficient. For the actual training, the speed of convergence may differ depending on the specific problem. However, the results in Section 5.5 suggest that the QGNNs showed a tendency to converge faster. Thus, overall, we expected the EDU-QGC QGNN models to require less computational cost compared to classical graph neural networks. However, the difference may be marginal depending on various factors such as the choice of the encoding method, convergence tendencies, and connectivity of the input graph.

### 5.6. Relationship between the Number of Trainable Variables and Model Performance

**Figure 23:**
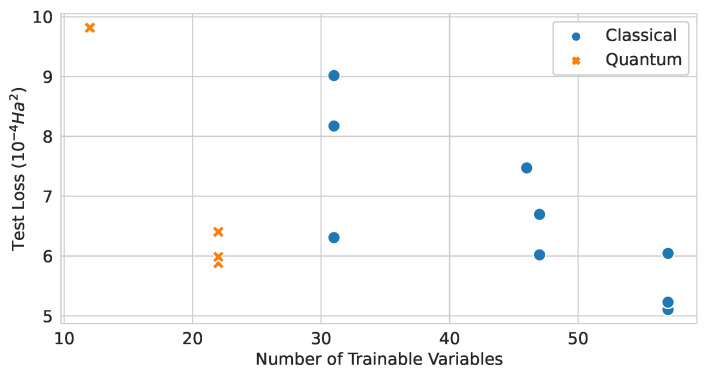
Relationship between the number of trainable variables and the test loss values of classical and quantum graph network models.

Runs with input node features of (Atomic Number and Number of Hydrogens) and three message passing or EDU-QGC layers were used to compare the relationship between the number of trainable variables and the test loss value. From the scatter plot in Figure 23, one can see a trend that the model performance improved as the number of trainable variables increased in general. When comparing the classical and quantum graph neural network models, quantum models are anticipated to show better performance compared to classical models for the same number of variables. This observation can be a signature that quantum models can provide the benefits of having less variables when aiming to build a model with a certain performance, such as faster and better training convergence on average. From this point of view, we can hypothesize that attempts to scale-up the quantum model are worthwhile.

## 6. Conclusions

This work aimed to give a brief review of graph neural networks for materials research and introduce their quantum analog by a novel quantum graph neural network model. The literature review showed that graph neural networks are being widely studied due to their natural and flexible representation of different systems and their resulting model performance. On the other hand, QGNN schemes are just emerging to be investigated for their potential advantage over classical models. Machine learning models of quantum, classical, and hybrid graph neural networks were trained for the QM9 dataset, and their performances in inferring the HOMO-LUMO gap were investigated. Even though the classical models performed similarly or marginally better than the quantum and hybrid models, there are certain aspects that would inspire further studies on QGNN models. When comparing the model performance measured by the number of trainable variables, the quantum models by the QGNN outperformed the classical models. The QGNNs tended to show faster convergence during training. Note that we claim no solid evidence for general quantum advantage, but report an indication that quantum models can achieve to a certain extent the performance benefits by requiring less trainable variables than classical models. Hopefully, these results will motivate more work in this converging field of quantum machine learning for novel materials design.

## Figures and Tables

**Table 1:** Node features and link features in the processed QM9 dataset.

	Feature	Explanation → Data Type
Nodes → Non-Hydrogen Atoms	Atomic Number	C/N/O/F → Integer
	Number of Bonded Hs	0~4 → Integer
	Aromaticity	True/False → Boolean
	Hybridization	$sp,sp^{2},sp^{3}\to$ Integer
Links → Bonds	Bond Type	Single/Aromatic/Double/Triple → Integer

**Table 2:** Quantum encoding methods. *z* is the atomic number; $n_{h}$ is the number of bonded hydrogen atoms; *a* is the aromaticity as True(1)/False(0); *h* is the hybridization type (sp: 1, $sp^{2}$: 2, $sp^{3}$: 3).

	Atomic Number	Atomic Number and Number of Hydrogens	Atomic Number, Aromaticity, and Hybridization
RY Rotation Angle	z=4→0, z=5,6,7→cos(−1/3)	$\frac{(2z-7)\pi }{4}$	$\frac{(2z-7)\pi }{4}$
RZ Rotation Angle	z=4,5→0, z=6→2π/3, z=7→−2π/3	$\frac{2\pi n_{h}}{5}$	$\frac{{\left(-1\right)}^{a}\left(2h-1\right)\pi }{6}$

**Table 3:** Test loss values of pure QGNNs with different encoding.

Encoding Method	Test MSE $\left(10^{-4}\;{\mathrm{Ha}}^{2}\right)$	Test RMSE $\left(10^{-2}\;\mathrm{Ha}\right)$	Test MAE $\left(10^{-2}\;\mathrm{Ha}\right)$
Atomic Number	6.3394	2.5178	1.9975
Atomic Number and Number of Hydrogens	5.8806	2.4250	1.8793
Atomic Number, Aromaticity, and Hybridization	7.1288	2.6700	2.0811

**Table 4:** Test loss values of pure QGNNs with different numbers of layers.

Number of Layers	Test MSE $\left(10^{-4}\;{\mathrm{Ha}}^{2}\right)$	Test RMSE $\left(10^{-2}\;\mathrm{Ha}\right)$	Test MAE $\left(10^{-2}\;\mathrm{Ha}\right)$
1	7.3759	2.7159	2.1400
2	6.5147	2.5524	2.0174
3	5.8806	2.4250	1.8793

**Table 5:** Test loss values of pure QGNNs with different readout functions.

Readout Function	Test MSE $\left(10^{-4}\;{\mathrm{Ha}}^{2}\right)$	Test RMSE $\left(10^{-2}\;\mathrm{Ha}\right)$	Test MAE $\left(10^{-2}\;\mathrm{Ha}\right)$
Local	5.8806	2.4250	1.8793
Global	6.4033	2.5305	1.9307

**Table 6:** Test loss values of pure QGNNs with different EDU-QGC architectures.

Model	Test MSE $\left(10^{-4}\;{\mathrm{Ha}}^{2}\right)$	Test RMSE $\left(10^{-2}\;\mathrm{Ha}\right)$	Test MAE $\left(10^{-2}\;\mathrm{Ha}\right)$
Default	5.8806	2.4250	1.8793
Simple	9.8162	3.1331	2.5045

**Table 7:** Test loss values of pure QGNNs with different modifications to the baseline model.

Model	Test MSE $\left(10^{-4}\;{\mathrm{Ha}}^{2}\right)$	Test RMSE $\left(10^{-2}\;\mathrm{Ha}\right)$	Test MAE $\left(10^{-2}\;\mathrm{Ha}\right)$
Baseline	5.8806	2.4250	1.8793
Baseline and Master Node	4.0372	2.0093	1.5106
Baseline and Re-Uploading	5.9863	2.4467	1.9379

**Table 8:** Test loss values of neural-network-assisted quantum encoding models and their quantum counterparts.

Node Features	Encoding Method	Test MSE $\left(10^{-4}\;{\mathrm{Ha}}^{2}\right)$	Test RMSE $\left(10^{-2}\;\mathrm{Ha}\right)$	Test MAE $\left(10^{-2}\;\mathrm{Ha}\right)$
Atomic Number	Fixed	5.8806	2.4250	1.8793
and Number of Hydrogens	Angle Extraction Network	5.3127	2.3049	1.7846
Atomic Number,	Fixed	7.1288	2.6700	2.0811
Aromaticity, and Hybridization	Angle Extraction Network	5.4597	2.3366	1.8097

**Table 9:** Test loss values of classical graph neural network models.

Architecture	GGNN Aggregation	Test MSE ($10^{-4}\;{\mathrm{Ha}}^{2}$)	Test RMSE $\left(10^{-2}\;\mathrm{Ha}\right)$	Test MAE $\left(10^{-2}\;\mathrm{Ha}\right)$
	Addition	6.0449	2.4586	1.9597
GGNN-GGNN-GGNN	Mean	5.1052	2.2595	1.7742
	Max	5.2289	2.2867	1.8053
	Addition	6.6965	2.5878	2.0311
GGNN-GGNN-GCN	Mean	6.0202	2.4536	1.9460
	Max	7.4732	2.7337	2.1468
	Addition	8.1737	2.8590	2.2079
GGNN-GCN-GCN	Mean	9.0177	3.0029	2.4088
	Max	6.3081	2.5116	2.0143

**Table 10:** Mean absolute error values of quantum, hybrid, and classical models on molecules with and without aromatic rings.

Model	With Aromatic Rings $\left(10^{-2}\;\mathrm{Ha}\right)$	Without Aromatic Rings $\left(10^{-2}\;\mathrm{Ha}\right)$
Quantum (Master Node)	1.9277	1.4128
Quantum (Baseline)	2.2509	1.7921
Hybrid	1.9323	1.7499
Classical	1.9820	1.7254

## Data Availability

The data are available upon request.

## Abbreviations

The following abbreviations are used in this manuscript:

EDU	Equivariantly diagonalizable unitary
EDU-QGC	Equivariantly diagonalizable unitary quantum graph circuit
EHQGC	Equivariant Hamiltonian quantum graph circuit
EQGC	Equivariant quantum graph circuit
GAN	Generative adversarial network
GCN	Graph convolutional network
GGNN	Gated graph neural network
HOMO-LUMO	Highest occupied molecular orbital-lowest unoccupied molecular orbital
MPNN	Message-passing neural network
NISQ	Noisy intermediate-scale quantum
PQC	Parameterized quantum circuit
QGNN	Quantum graph neural network
QNN	Quantum neural network
VAE	Variational autoencoder
